# Quantification of LDL-Cholesterol Corrected for Molar Concentration of Lipoprotein(a)

**DOI:** 10.1007/s10557-022-07407-y

**Published:** 2022-11-26

**Authors:** Robert S. Rosenson, J. Antonio G. López, Maria Laura Monsalvo, You Wu, Huei Wang, Santica M. Marcovina

**Affiliations:** 1 Metabolism and Lipids Unit, Icahn School of Medicine at Mount Sinai, One Gustave L. Levy Place, Hospital Box, 1030, NY 10029 New York, USA; 2 Global Development, Amgen Inc., Thousand Oaks, CA USA; 3 Medpace Reference Laboratories, Cincinnati, OH USA

**Keywords:** Apo(a) isoform, Apolipoprotein B, Cardiovascular risk, LDL-cholesterol, Lipoprotein(a), Methods

## Abstract

**Purpose:**

Cholesterol in lipoprotein(a) [Lp(a)-C] is commonly estimated as 30% of the measured Lp(a) mass. However, difficulties in the accurate measurement of Lp(a) mass, along with the inaccuracy of the 30% assumption, produce erroneous values when LDL-C is corrected for Lp(a) [LDL-C_**Lp(a)corr**_]. Our aim was to develop a new formula for LDL-C_**Lp(a)corr**_ to reduce this error.

**Methods:**

We developed a new formula to calculate Lp(a)-C from the molar measurement of Lp(a), which is Lp(a) nmol/L × 0.077 = Lp(a)-C mg/dL. The calculated Lp(a)-C is subtracted from LDL-C to obtain LDL-C_**Lp(a)corr.**_ The results obtained with our novel formula versus the conventional formula were compared in 440 samples from 239 participants enrolled in the BANTING study.

**Results:**

With the conventional formula, approximately 7% of samples with low LDL-C resulted in negative LDL-C_**Lp(a)corr**_ values. With the new formula, no negative LDL-C_**Lp(a)corr**_ values occurred. Among groups with the highest Lp(a)/apoB ratio (*p* < 0.001) and smaller apolipoprotein(a) isoform size (*p* < 0.006), LDL-C_**Lp(a)corr**_ was significantly underestimated by the conventional formula, which may result in the undertreatment of some patients.

**Conclusion:**

The new formula provides more reliable estimates of LDL-C_**Lp(a)corr**_ than the conventional formula.

**Trial registration:** ClinicalTrials.gov NCT02739984.

**Supplementary Information:**

The online version contains supplementary material available at 10.1007/s10557-022-07407-y.

## Introduction

Lipoprotein(a) [Lp(a)] is an apolipoprotein B (apoB)-containing lipoprotein particle formed by low-density lipoprotein (LDL) but characterized by the presence of a highly glycosylated, highly polymorphic in size protein termed apolipoprotein(a) [apo(a)], linked by a single disulfide bond to the apoB component of LDL. Elevated Lp(a) is a risk factor for atherosclerotic cardiovascular disease (ASCVD) and aortic valve stenosis even at very low levels of LDL-C, and the recent European Atherosclerosis Society consensus statement considers including Lp(a) in global risk estimation [1]. The quantification of LDL-cholesterol (LDL-C), independent of the method used for its determination, also includes the cholesterol content in Lp(a). In the past several years, there has been a growing interest in quantifying the contribution of Lp(a)-cholesterol [Lp(a)-C] to LDL-C. Due to the lack of commercially available methods to directly measure cholesterol in Lp(a), different studies have estimated Lp(a)-C as a fixed percentage of Lp(a) total mass. Based on compositional studies performed on Lp(a) isolated from few individuals, the cholesterol content of Lp(a) was found to represent about 30% of the total Lp(a) mass [2]. Despite the inaccuracy of the laboratory methods used to measure Lp(a) mass in plasma samples and the uncertainty about the percentage of cholesterol in Lp(a) particles, Lp(a)-C is calculated to be 30% of the total Lp(a) mass, and this obtained value is then subtracted from LDL-C to calculate Lp(a)-corrected LDL-C [LDL-C_**Lp(a)corr**_] [3]. This approach involves multiple assumptions that compound the errors, particularly in individuals with low LDL-C and high Lp(a) [3]. These inaccurate estimates preclude meaningful data analysis and accurate interpretation of Lp(a) versus non-Lp(a) LDL-C in clinical trials of lipid therapies that also impact Lp(a), including selective therapies for Lp(a) lowering. Additionally, these erroneous values, magnified in individuals with low LDL-C and high Lp(a) levels, provide misleading data for clinicians, investigators, and patients. Results of a recent study performed on a large number of patients from the Very Large Database of Lipids showed that the conventional estimate of Lp(a)-C of 30% of Lp(a) mass overestimates Lp(a)-C with progressively higher Lp(a) levels, and consequently underestimates LDL-C_**Lp(a)corr**_ [4].

Here, we present a novel approach, the Rosenson-Marcovina formula, to calculate Lp(a)-C to express LDL-C_**Lp(a)corr**_ and compare it with the result obtained with the commonly used approach, in which Lp(a)-C is calculated as 30% of the total plasma Lp(a) mass.

### Methods

We used data from 239 participants with 440 samples enrolled in BANTING, a placebo-controlled trial of evolocumab in patients with type 2 diabetes and high cardiovascular risk (NCT02739984) [5]. The demographic and clinical characteristics of the study population have been previously presented in detail [5]. Samples without missing Lp(a) and lipid data on day 1 and week 12 were used in this analysis. An institutional review board or independent ethics committee reviewed and approved the study protocol and the amendment at each study center. Participants provided written informed consent before any screening procedures. This study was conducted in accordance with the International Council for Harmonisation Good Clinical Practice regulations/guidelines.

Molar concentrations of Lp(a) were determined by a particle-enhanced turbidimetric assay using a Roche reagent on a Roche c502 instrument. The assay calibrator is traceable to the World Health Organization (WHO)/International Federation of Clinical Chemistry and Laboratory Medicine (IFCC) reference material SRM-2B, and the method has been demonstrated to measure Lp(a) with minimal impact of apo(a) size variation [6].

Apo(a) isoform size was determined using a sensitive agarose gel electrophoresis method, in which the migration in the gel is proportional to the number of apo(a) kringle IV (KIV) motifs in the samples [7]. For analysis, data were divided into tertiles of predominant apo(a) isoform size expressed in the number of KIV motifs.

Total apoB was determined using Siemens reagent on a Siemens BNII nephelometer. The assay calibrator is traceable to the WHO/IFCC Reference Material SP3-08, and the results are reported in mg/dL.

Because there is 1 mol of apo(a) and 1 mol of apoB in each Lp(a) particle, the results obtained by the molar determination of Lp(a) represent either the mole of apo(a) or the mole of apoB. Unlike apo(a), apoB has a constant mass, and therefore, the concentration of apoB in Lp(a) can be accurately converted from nmol/L to mg/dL. Although slightly different molecular weights for apoB have been reported, in our formula, we used the molecular weight of 513,000 kDa obtained by amino acid analysis, which is considered an accurate method for determining the molecular weight of proteins [8].

Samples from 50,059 individuals from the general population, participants in different clinical trials, as well as from patients with different conditions, including diabetes and ASCVD, were sequentially analyzed between 1990 and 2018 at the Northwest Lipid Research Laboratory, University of Washington by density gradient ultracentrifugation (DGUC) [9]. After separation of lipoproteins by flotation characteristics, 38 fractions were obtained using a fraction collector. After combining the fractions of the narrow peak LDL, thus excluding intermediate-density lipoprotein and Lp(a) fractions, cholesterol and apoB were measured. By calculating the relative flotation rate of the peak LDL, the 50,059 individuals were characterized as having predominantly small, dense LDL, intermediate-density LDL, and large, buoyant LDL. For each of the three categories, we calculated the mean LDL-C to LDL-apoB ratio, which was 1.385 for small, dense LDL; 1.510 for intermediate-density LDL; and 1.545 for large, buoyant LDL; with an overall mean ratio of 1.497 (Online Resource 1, unpublished data). Assuming that the LDL particles in Lp(a) have the same cholesterol to apoB ratio as circulating LDL particles, this ratio was applied to calculate the cholesterol content of Lp(a). Therefore, Lp(a)-C (mg/dL) = Lp(a) nmol/L × 0.0513 (molecular weight of apoB) × 1.497 (mean cholesterol to apoB ratio) or more simply, Lp(a)-C (mg/dL) = Lp(a) nmol/L × 0.077. To evaluate the effects of varying cholesterol content in LDL particles, Lp(a)-C was also calculated using the mean cholesterol to apoB ratio of 1.545, 1.510, and 1.385, obtained for large, buoyant LDL, intermediate LDL, and small, dense LDL, respectively.

Using a reflexive approach, we directly measured LDL-C by beta quantification when LDL-C levels were < 40 mg/dL or triglycerides ≥ 400 mg/dL and used the NIH-2 equation for LDL-C levels ≥ 40 mg/dL [10]. All summaries were performed for all records without considering study visits and treatment groups.

### Statistical Analysis

LDL-C_**Lp(a)corr**_ values calculated from both the conventional estimate and the Rosenson-Marcovina formula were summarized by LDL-C decile categories. Similarly, LDL-C_**Lp(a)corr**_ values calculated from both methods were summarized separately based on the tertile categories of the percentage of Lp(a) to total apoB categories and apo(a) predominant isoform size. To calculate the molar percentage of Lp(a) to total apoB, the mass of total apoB was converted to molar concentration using the molecular weight of apoB of 513 kg/mol. Descriptive statistics included mean with standard deviation and median with interquartile range. The comparison between the two approaches to calculating LDL-C_**Lp(a)corr**_ was performed using a mixed model, with the estimate approach as a fixed effect and patient as a random effect.

## Results

**Fig. 1:**
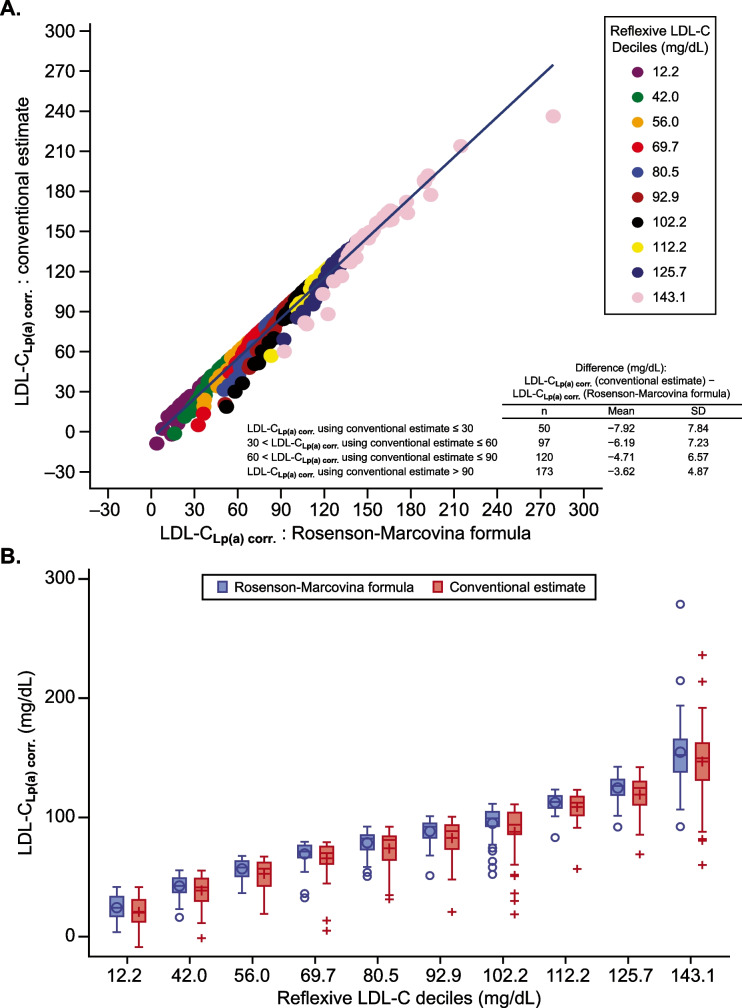
Conventional estimate versus Rosenson-Marcovina formula: dot plot with regression line and boxplot of reflexive LDL-C deciles for the overall population. LDL-C, low-density lipoprotein cholesterol; LDL-C_**Lp(a)corr, **_lipoprotein(a)-corrected low-density lipoprotein cholesterol; SD, standard deviation

Overall, LDL-C levels ranged from 12.2 to 346.7 mg/dL (in the placebo and evolocumab groups combined); Lp(a) concentrations ranged from 2.9 to 884.2 nmol/L. Generally, the variation in LDL-C_**Lp(a)corr**_ increased in parallel with LDL-C level and was consistent across both the conventional estimate and the Rosenson-Marcovina formula. Variations in LDL-C_**Lp(a)corr**_ using the conventional approach were larger than variations in the estimates using the Rosenson-Marcovina formula (Fig. 1A, B). The formula-based LDL-C_**Lp(a)corr**_ values were higher than that obtained with the conventional approach. Based on LDL-C_**Lp(a)corr**_ obtained by the conventional estimate cutoff points of ≤ 30, > 30 to ≤ 60, > 60 to ≤ 90, and > 90 mg/dL, the mean difference between the two estimates decreased when the LDL-C_**Lp(a)corr**_ cutoff points increased. The variation in the difference was larger in the groups with lower LDL-C_**Lp(a)corr**_ obtained by the conventional estimate. Additionally, 4.6% of measurements with LDL-C levels ≤ 12.2 mg/dL and 2.3% of measurements with LDL-C levels between > 12.2 and < 42 mg/dL had negative LDL-C_**Lp(a)corr**_ values (Fig. 1A). When we evaluated different LDL-C/apoB ratios, the coefficients of variation (CVs) for small and intermediate LDL were very close (9.6% and 8.4%, respectively) while a CV of 15.5% was observed for large, buoyant LDL, indicating a larger variability in the cholesterol to apoB ratio in large LDL particles (Online Resource 1). When we evaluated differences in LDL-C_**Lp(a)corr**_ obtained by the three different ratios, no substantial differences in results were observed either overall or by LDL-C decile (Online Resource 2 and Online Resource 3).

In the group with the lowest number of apo(a) KIV, the conventional estimate significantly underestimated LDL-C_**Lp(a)corr**_ (*p* = 0.006) compared with the Rosenson-Marcovina formula (Table 1). No significant difference between the two estimates was observed with a higher number of KIV. Among the groups with the highest percentage of Lp(a) to total apoB, the conventional estimate significantly underestimated LDL-C_**Lp(a)corr**_ (*p* < 0.001; Table 2), whereas no significant differences between the two methods were observed when the percentage of Lp(a) to total apoB was < 6.5%.

**Table 1:** Mean (SD) LDL-C_**Lp(a)corr**_ values for conventional estimate and Rosenson-Marcovina formula by apo(a) isoform size tertiles

Number of apo(a) kringle IV tertiles	Number of measurements	Conversion	Mean (SD) LDL-C_**Lp(a)corr**_	*p*-value*
11–19	159	Rosenson-Marcovina	73.4 (36.9)	0.006
		Conventional	64.6 (38.1)	
20–25	144	Rosenson-Marcovina	85.2 (39.4)	0.219
		Conventional	81.1 (37.8)	
26–37	137	Rosenson-Marcovina	97.4 (39.4)	0.635
		Conventional	95.9 (39.2)	

^*^Mixed-effects model. *apo(a)*, apolipoprotein(a); *LDL-C*_***Lp(a)corr***_*,* lipoprotein(a)-corrected low-density lipoprotein cholesterol; *SD*, standard deviation

**Table 2:** Mean (SD) LDL-C_**Lp(a)corr**_ values and statistical comparison for conventional estimate and Rosenson-Marcovina formula by percentage of Lp(a) to total apoB tertiles

Percentage of Lp(a) to total apoB (%)	Number of measurements	Conversion	Mean (SD) LDL-C_**Lp(a)corr**_	*p*-value*
0.1 to < 1.2	146	Rosenson-Marcovina	93.7 (37.3)	0.888
		Conventional	93.3 (37.3)	
1.2 to < 6.5	147	Rosenson-Marcovina	89.5 (38.4)	0.302
		Conventional	86.7 (37.5)	
6.5 to 48.9	147	Rosenson-Marcovina	71.1 (39.7)	< 0.001
		Conventional	59.4 (38.2)	

^*^Mixed-effects model. *apoB*, apolipoprotein B; *LDL-C*_***Lp(a)corr***_, lipoprotein(a)-corrected low-density lipoprotein cholesterol; *Lp(a)*, lipoprotein(a); *SD*, standard deviation

## Discussion

Due to the multiple assumptions inherent in estimating the cholesterol content of Lp(a), the American Heart Association Scientific Statement on Lp(a) suggested that clinicians calculate the proportion of apoB-containing lipoproteins ascribable to Lp(a) by dividing the molar concentration of Lp(a) by the total plasma apoB converted to nmol/L [11]. While it is true that apoB concentration may be a better predictor than LDL-C in estimating the incident and residual cardiovascular risk [11], at present, LDL-C is the major therapeutic target for the prevention of ASCVD. Therefore, our aim was to develop a formula that more accurately calculates Lp(a)-C to be subtracted from LDL-C. With the introduction of drugs that potently lower LDL-C, there has been a strong interest in recent years in determining the contribution of Lp(a)-C to LDL-C and its impact on cardiovascular disease risk assessment and treatment. The need to correctly estimate the proportion of LDL-C that is Lp(a)-C is particularly important in patients with high Lp(a) levels and low LDL-C levels in whom the conventional estimate of Lp(a)-C may result in negative LDL-C values.

Currently, no reliable commercial methods are available in clinical laboratories for directly measuring Lp(a)-C, and the most common approach used in the literature has been to estimate Lp(a)-C as being 30% of the measured Lp(a) mass. However, this estimate has several problems that may impact its reliability. The lack of traceability of Lp(a) mass to a common reference material and the impact of apo(a) size variation result in significant differences in Lp(a) mass values determined by the different commercial methods. These methodological problems are further compounded by the assumption that the immunochemically determined Lp(a) mass in plasma samples is the same as the mass obtained by compositional studies on isolated Lp(a) from where cholesterol was determined to be 30% of the Lp(a) mass. The results of a recent study performed on a large number of individuals have demonstrated that Lp(a)-C concentration is overestimated by considering Lp(a)-C as 30% of Lp(a) mass [4], thus resulting in lower LDL-C_**Lp(a)corr**_.

We have presented here a novel approach to estimate Lp(a)-C based on molar measurement of Lp(a) performed by a method minimally affected by the size variation of apo(a). We have then calculated the ratio between cholesterol and apoB in LDL determined in 50,059 samples where LDL-C and apoB were measured by DGUC in the narrow LDL peak fraction. The average LDL-C/apoB ratio (1.497), obtained in samples with predominantly small, intermediate, or large LDL particles, was used in our formula to calculate Lp(a)-C. To apply this average ratio to the determination of Lp(a)-C required the assumption that the LDL-like particles in Lp(a) have a similar mean ratio between cholesterol and apoB as the circulating LDL. No large studies have been performed so far to evaluate the distribution of lipids in Lp(a), but it would be difficult to expect that the amount of cholesterol in Lp(a) particles is so different from that in LDL to significantly impact the mean cholesterol to apoB ratio used in our formula. Additionally, when we calculated LDL-C_**Lp(a)corr**_ using the relative ratios for small, intermediate, and large LDL instead of the overall ratio, no substantial difference in results was observed, neither overall nor by LDL-C deciles. However, we still need to consider that our formula may not correctly estimate Lp(a)-C in samples with very small or very large amounts of cholesterol. Yeang et al. recently reported the development of a method for directly quantifying cholesterol in Lp(a) particles [12, 13]. By this method, Lp(a)-C is measured enzymatically following isolation of Lp(a) particles by an apo(a)-specific monoclonal antibody linked to magnetic beads. Analyses were performed on samples from 29 subjects collected at three different visits. Overall, Lp(a)-C ranged from 0.6 to 35.0 mg/dL and the percent of Lp(a)-C to Lp(a) mass varied from 5.8 to 57.3%. This extreme variation in the content of Lp(a) cholesterol measured by this method is by far higher than that observed in LDL particles. Considering that these data were obtained on only 29 patients with high Lp(a), 21 undergoing Lp(a)-lowering therapy, and nine controls, the variability of cholesterol in Lp(a) particles would need to be assessed in a larger and more diverse population. In addition, this method is not at present commercially available, and therefore, a direct comparison of results obtained by our formula and by the direct measurement of Lp(a)-C is not feasible.

Based on our formula, we have shown that Lp(a)-C is lower than that obtained by the conventional 30% estimate, thus resulting in higher LDL-C_**Lp(a)corr**_. Using the conventional calculation, approximately 7% of samples with low LDL-C levels resulted in negative LDL-C_**Lp(a)corr**_ values. In contrast, no negative levels were obtained using the Rosenson-Marcovina formula.

We found that the group with the smallest apo(a) isoform size had significantly lower LDL-C_**Lp(a)corr**_ with the conventional approach than with the Rosenson-Marcovina formula.

Additionally, we found that among the groups with the highest percentage of Lp(a) to total apoB (6.5 to 48.9%), the conventional approach significantly underestimated LDL-C_**Lp(a)corr**_.

Taken together, the results indicate that in cases of high Lp(a) levels, the conventional approach overestimates Lp(a)-C, thus resulting in underestimation of LDL-C_**Lp(a)corr**_. This underestimation is clinically relevant because many patients with high Lp(a) levels may be undertreated for LDL-C following national cholesterol and international dyslipidemia guidelines [14, 15].

In summary, using the formula Lp(a) nmol/L × 0.077 to calculate Lp(a)-C in mg/dL and subtracting it from LDL-C provides a more reliable estimate of the relative atherogenic contribution of LDL-C_**Lp(a)corr**_ versus the estimate based on Lp(a)-C being 30% of Lp(a) total mass. Our approach, based on the calculation of cholesterol in Lp(a) by the molar determination of Lp(a) and by a well-supported mean ratio between cholesterol and apoB, provides a more precise measure of LDL-C_**Lp(a)corr**_. Additionally, a more accurate calculation of Lp(a)-C avoids the spurious negative LDL-C values observed using the conventional estimate. Unlike for Lp(a), where a “gold standard” ELISA is available [6], a “gold standard” method for determination of Lp(a)-C is not presently available. Therefore, a direct evaluation of the accuracy of our formula as well as the accuracy of the direct method proposed by Yeang et al. [12], is not possible. In conclusion, our formula can be easily used in clinical laboratories to reliably calculate LDL-C_**Lp(a)corr**_ without additional cost, even though a validation of this proposed approach, performed on a larger number of individuals with and without diabetes and with and without ASCVD, needs to be performed to ascertain its clinical usefulness.

### Supplementary Information

Below is the link to the electronic supplementary material.

Supplementary file1 (PDF 128 KB)

## Data Availability

Qualified researchers may request data from Amgen clinical studies. Complete details are available at www.amgen.com/datasharing.
