# Supplementary material for: Quantification of LDL-Cholesterol Corrected for Molar Concentration of Lipoprotein(a)
Source: Cardiovasc Drugs Ther. 2022 Nov 26;38(1):191–7. doi: 10.1007/s10557-022-07407-y (PMC10876802; doi:10.1007/s10557-022-07407-y)
Supplement: Supplementary file 1 — Supplementary file1 (PDF 128 KB) [file 10557_2022_7407_MOESM1_ESM.pdf]

Supplemental Material

Quantification of LDL-Cholesterol Corrected for Molar Concentration of Lipoprotein(a). *Cardiovascular Drugs and Therapy*. Robert S. Rosenson,[a] J. Antonio G. López, Maria Laura Monsalvo, You Wu, Huei Wang, Santica M. Marcovina

[a]Metabolism and Lipids Unit, Icahn School of Medicine at Mount Sinai, One Gustave L. Levy Place, Hospital Box 1030, New York, NY 10029, USA. Email: robert.rosenson@mssm.edu.

Online Resource 1 Ratio of cholesterol to apoB in LDL particles isolated by density gradient ultracentrifugation

LDL particles	Average ratio	Standard deviation	Sample number	CV, %
Large, buoyant	1.545	0.239	22,080	15.47
Intermediate	1.510	0.127	16,536	8.41
Small, dense	1.385	0.133	11,443	9.60
Overall	1.497	0.197	50,059	

apoB, apolipoprotein B; CV, coefficient of variation; LDL, low-density lipoprotein.

Online Resource 2 Summary of LDL-$C_{\mathbf{Lp(a)corr}}$ in mg/dL based on different ratios of cholesterol to apoB

Ratio	Number of observations	Mean (SD) LDL-$C_{\mathbf{Lp(a)corr}}$ (mg/dL)
1.55	440	84.47 (39.67)
1.5	440	84.73 (39.66)
1.4	440	85.26 (39.65)

Overall *p*-value=0.90 calculated from a mixed-effects model with the estimate approach as a fixed effect and patient as a random effect. apoB, apolipoprotein B; LDL-CLp(a)corr, Lp(a)-corrected LDL-C; SD, standard deviation.

Online Resource 3 Comparison of conventional estimate and Rosenson-Marcovina formula with different ratios of cholesterol to apoB by reflexive LDL-C deciles

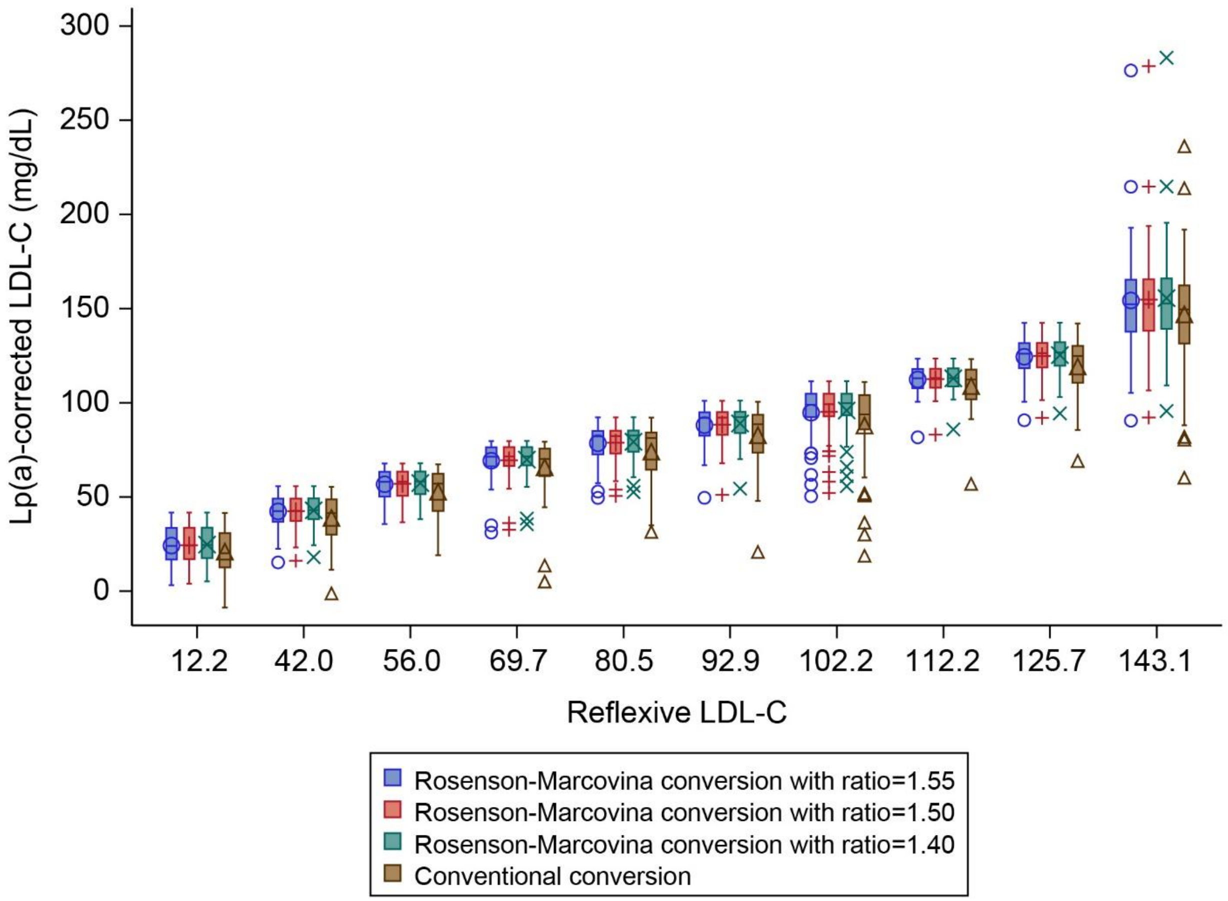

apoB, apolipoprotein B; LDL-C, low-density lipoprotein cholesterol; Lp(a), lipoprotein a.
